# The informational roles and psychological health of members of 10 oncology multidisciplinary teams in the UK

**DOI:** 10.1038/sj.bjc.6602816

**Published:** 2005-10-18

**Authors:** S Catt, L Fallowfield, V Jenkins, C Langridge, A Cox

**Affiliations:** 1 Cancer Research UK, Psychosocial Oncology Group, Brighton & Sussex Medical School, University of Sussex, Falmer, Brighton BN1 9QG, UK

**Keywords:** multidisciplinary teams, psychological well being, burnout

## Abstract

We report here the different roles undertaken by the members of 10 multidisciplinary cancer teams in conveying information to patients during their care. Team members completed an Informational Roles Questionnaire measuring an individual's perception of their major role and that of their colleagues in giving information to patients. They also completed two standard psychological health measures, the General Health Questionnaire and Maslach Burnout Inventory. The information giving roles of the surgeon, oncologist, radiologist and clinical nurse specialist were well recognised by their colleagues; however, other team members' roles were more ambiguous and less well understood. The clinical nurse specialist provided the broadest information coverage for patients. Few professional groups regularly informed patients about clinical trials and family history and the clinical nurse specialist was often the only person to deal with patients' sexual well being, consequently these areas are likely to receive poor coverage. Probable psychiatric morbidity (GHQ⩾4) in teams ranged from 5 to 27%. High levels of emotional exhaustion were particularly apparent in team leaders and nurses and feelings of low levels of personal accomplishment were prevalent in the histopathologists and radiologists. Putative benefits to patients and healthcare professionals from multidisciplinary team working may not be realised without investment in team training.

The multidisciplinary team (MDT) evolved in response to the increasing complexities of patient care, with each discipline contributing his/her particular skills and knowledge for the benefit of the patient (Hall and Weaver, 2001). Since the publication of the Calman–Hine report concerning reorganisation of cancer services in the UK, many centres have adopted a MDT approach with the aim of providing the patient with the best care (Calman and Hine, 1995). In the Improving Outcomes Guidance (NHS Executive, 1999, 2002, 2004) the core professions typically involved in providing multidisciplinary care to patients with cancer are surgery, oncology, pathology, radiology and nursing. Additionally it may include, palliative care, psychiatry/psychology, genetics and plastic surgery. The concept of a MDT should not merely be a group of professionals who work essentially independently and occasionally liase with one another (Miller *et al*, 2001). Effective interprofessional team working needs the evolution of a shared team culture, open communication, mutual respect for all the practitioners and equal value to be placed on their contribution to current team practices (Freeman *et al*, 2000). It is suggested that this can only be achieved when each member of the team understands the others' contributions to care, as well as understanding how and why they practise in the way they do and this requires group process to be nurtured (Miller *et al*, 2001). The expectation is that successful multidisciplinary teamwork is beneficial both for the patient and members of the team.

Early retrospective reviews of cancer registries (Junor *et al*, 1994; Sainsbury *et al*, 1995) concluded survival prospects for patients were better when a multidisciplinary approach to treatment and care had been employed. Similarly combined modality therapy was emerging as the treatment of choice for patients with breast cancer leading the way to multidisciplinary management (Hortobagyi, 1994). An examination of the relationship between cancer teams and the quality of care delivered showed MDT working benefited patients through improved access to, and use of, standardised and up-to-date therapy (Landheer *et al*, 2001). Papers outline the necessity of a multidisciplinary approach for optimizing outcome in patients with cancer (Van Laethem *et al*, 2001; Blumberg and Ramanathan, 2002) and some evidence exists to show that specialized multidisciplinary units increase the efficacy and efficiency of the management of patients with cancer (Shankar *et al*, 2001; Soriano *et al*, 2002; Haward *et al*, 2003). However, quality of clinical care and team effectiveness has been shown to be related to team composition, working methods and workloads (Haward *et al*, 2003).

Ramirez *et al* (1996) identified poor communication and a lack of management skills training as major factors leading to burnout and psychiatric morbidity in UK consultants. The Maslach Burnout Inventory (MBI) was used to assess the three components of ‘burnout’ (emotional exhaustion, depersonalisation and low personal accomplishment) and the General Health Questionnaire (GHQ-12) to measure the consultants' psychiatric morbidity and results showed that radiologists exhibited the highest level of burnout in terms of low personal accomplishment while 27% of consultants had a GHQ-12 score of 4 or more (indicating likely psychiatric morbidity). These data have been recently up-dated with longitudinal follow-up results showing that the prevalence of psychiatric morbidity in these UK consultants had risen to 32% and that emotional exhaustion had increased from 32% in 1994 to 40% in 2002 (Taylor *et al*, 2005). The authors relate this decline in mental health to increased job stress without a comparable increase in job satisfaction and noted that this was especially marked in clinical and surgical oncologists. ‘Burnout’ symptoms can include dysfunctional attitudes and behaviours towards colleagues, which in turn will have a detrimental effect on team functioning, communication and patient care.

However, research in the primary healthcare setting has shown that those working within a supportive, well-functioning team, benefited from better mental health and increased team effectiveness (Carter and West, 1999). Complex tasks were accomplished more easily when professionals within the healthcare teams had clear goals, were cooperative and mutually supportive of one another and aware of each other's role (Firth-Cozens, 1999; Payne, 1999). An extensive study covering a 3-year period, focusing on three types of MDT (100 primary healthcare teams, 113 community mental healthcare teams and 193 secondary healthcare teams) showed that better team functioning was associated with better mental health (Borrill *et al*, 2000). This finding has been reiterated by Haward *et al* (2003) who reported a beneficial effect of team working on mental health in breast cancer teams, where prevalence for minor psychiatric morbidity measured using the GHQ-12 was as low as 15.7% across all teams, a figure below that of 18% given for the UK general population (Taylor *et al*, 1995). These findings contrast with rates of 28–32% given for a cohort of UK consultants (Ramirez *et al*, 1996; Taylor *et al*, 2005) and those of past data for NHS trust staff in general where an incidence of 27% has been reported (Wall *et al*, 1997). Mullarky *et al* (1999) have reported incidences in community mental health teams, secondary-care teams and primary healthcare teams of 26, 23 and 22%, respectively.

The Improving Outcomes Guidance (NHS Executive, 1999, 2002, 2004) makes explicit recommendations about communication between healthcare professionals within a MDT and between themselves and patients. However, there is little available data on the advantages and disadvantages of a MDT approach to communication within cancer care. Anecdotal evidence from hospitals and medical defence organisations suggests poor communication can result in complaints and litigation. Reports from the USA demonstrate that a considerable proportion of lawsuits originate from misunderstandings, and not treatment errors (Gorney, 1999; Krause *et al*, 2001). There is increasing evidence that effective communication is a critical means by which doctors can assist their patients to achieve the best outcomes (Boyle *et al*, 2004). Audits have been performed to determine the accuracy and consistency of information delivered to breast cancer patients within the healthcare system (Hughes and Bradburn, 1996; Harris, 1997). Both reports showed that the provision and consistency of information presented to the patient differed within the health team. Patients in these surveys stated that they received insufficient information and noted that there was poor communication between the healthcare professionals. Early work concerning communication within breast cancer teams revealed a lack of interdisciplinary awareness of team members' roles and an inability to identify the team leader by a significant minority of MDT members (Jenkins *et al*, 2001).

We present results on healthcare professionals who participated in a Cancer Research UK funded study looking at MDT communication. The aims of the assessments carried out with MDT members were (1) to describe team members information-giving roles, (2) to identify the strengths and weaknesses of communication within oncology MDTs in terms of members information giving roles and (3) to assess team members mental health and compare it with previously published data.

## MATERIALS AND METHODS

Five breast, three colorectal and two gynaecology multidisciplinary cancer teams ranging in size from eight to 21 members, recruited from England, Scotland and Wales took part in the study. Each team identified their own regular members; the only stipulation made by the researchers was that all must be attendees at the weekly MDT meetings. The professional make-up of each team is given in Table 1. Team members read an information sheet and gave written consent before participating. The study had multiple regional and local ethical approvals.

Team members completed the Informational Roles Questionnaire (IRQ) (Jenkins *et al*, 2001) which measures healthcare professionals perceptions of their own role and their awareness of their colleagues' roles in providing information to the patient during their treatment and care. The list of areas covered varied by cancer site but typically included: talking about diagnostic tests, giving the diagnosis of cancer, discussing surgery, radiotherapy, chemotherapy, prognosis, clinical trials, family history and the provision of written materials. Team members were also asked to indicate whether they and which of their colleagues have a regular role in discussing patient problems in each of five areas of psychosocial concern: (1) physical, (2) functional, (3) sexual, (4) social and (5) emotional well being. They also completed two measures of psychological well being; the 12-item GHQ-12 and the MBI. The GHQ-12 is a self-report questionnaire specifically designed to screen for nonpsychotic psychiatric disorder, it is well validated and has been widely used in samples of healthcare professionals. It measures 12 symptoms of minor psychiatric morbidity (e.g., depression, loss of confidence, sleep disturbance) and these are rated according to whether they have been experienced ‘not at all’, ‘the same as usual’, ‘rather more than usual’, or ‘much more than usual’ in the past few weeks. Individuals scoring above a threshold of ⩾4 are highly likely to merit a diagnosis of clinical anxiety or depression according to studies validating the GHQ-12 against standardized psychiatric interviews (Goldberg and Williams, 1988). The MBI (Maslach *et al*, 1996) is also a self-report questionnaire used to measure the effect that working closely with people in an emotionally demanding role has on a person's mental health. The inventory has three dimensions each of which are reported on:

- *Emotional Exhaustion* (*EE*) – the degree to which a person feels emotionally overextended and exhausted by their work.
- *Depersonalisation* (*DP*) – the degree to which a person has developed feelings of indifference and cynical attitudes toward recipients of the care, treatment, instruction or service they provide to others in their work.
- *Personal Accomplishment* (*PA*) – the degree to which a person gains feelings of competence and successful achievement in their work.

## RESULTS

Results from the IRQ are expressed in tabulation form so that percentage of agreement about what information-giving areas are covered by a role can be examined. Responses to the IRQ allow two views to be represented for each information-giving role and these two views can be compared with each other. The two views of interest are (1) what individuals within a specialist group, for example, surgeons, agree they talk to patients about, referred to as the ‘speciality view’ and (2) what the rest of the team members believe the surgeons talk to the patients about, referred to as the ‘colleagues view’. Consistency within a professional group can be scrutinised as well as looking for congruence between other team member's expectations of a role and the picture given by those individuals who actually carry out the job. Additionally, tabulation allows examination across the speciality views, which can reveal overlaps and gaps in information giving. Tables 2a and 2b show the information-giving roles by professional group comparing each individual speciality's view of their role with the view of their informational role held by the other team members. In Table 2a professions that are well recognised by team members are presented. For example, all of the surgeons agree they discuss prognosis and 96% of their colleagues expected this, conversely few surgeons gave out leaflets but this was consistent with few of their colleagues believing they did. Table 2b on the other hand gives examples of specialities that are more ambiguous and less well understood by team members. An example includes the fact that all of the clinic nurses regularly discuss physical, functional, social and emotional well being with patients yet few of their colleagues showed any awareness of this.

It should be noted that results are purely descriptive and that statistical testing of consistency between a ‘speciality view’ and the ‘colleagues view’ was not considered appropriate due to the small numbers and multiple comparisons that would be involved in such an analysis.

Table 3 shows the number of different professions regularly covering each information area. In all, 12 out of 14 professions regularly talked to patients about physical well being, in contrast only three and four, respectively, recognised the discussion of clinical trials and family history as their responsibility. Although five professions provided information on sexual well being only one afforded guaranteed access to all patients and this was the clinical nurse specialist. Table 3 also shows that the clinical nurse specialists covered the broadest range of information areas 11/14, as well as regularly giving out information leaflets.

Table 4 shows the proportion of members scoring above the threshold of ⩾4 on the GHQ-12 and the percentage of members with high levels of ‘burnout’ on each subscale of the MBI for each team and gives a breakdown by speciality. It should be noted that some specialities are not presented in Table 4 in order that anonymity is preserved.

## DISCUSSION

In terms of information coverage, results showed that the surgeons and oncologists had primary responsibilities for conveying information to patients about their investigations, results including ‘bad news’, treatment options (surgery, radio-, chemo- and hormone therapy) and prognosis. Multiple professional groups were shown to regularly discuss these areas with the patient. It is not unusual for patients to seek the same piece of information from a series of health professionals, because they forget, because they do not understand the first time around, because they want to be sure, and this underlines the point that it is crucial for members within teams to be sure they are giving the same message, that there is consistency within and across information giving. Further, these vital areas were always backed up by the role of the clinical nurse specialist who it was revealed covered the largest number of information areas identifying them as the thread that runs throughout the patients care in these cancer teams. However, when it came to other information areas, coverage was not always so comprehensive for patients. Interestingly, the two areas where there was least consensus about coverage among the clinical nurse specialists, clinical trials and family history, were the same two areas recognised by the least number of professional groups (3–4/14) as part of their information-giving role. These factors coupled together suggest there is potential for weakness in coverage of clinical trials and family history, particularly as some of the specialties taking responsibility for providing this information, for example, palliative care physicians, research nurses, do not routinely see all patients. The clinical nurse specialists were shown to be responsible for covering the psychosocial well being of patients, a role that has traditionally been a nursing domain. However, in many instances in these teams there was no guaranteed fall back for covering these areas were the clinical nurse specialist to be off sick, on annual leave or otherwise unavailable. The potentially weaker areas of information coverage, clinical trials, family history and sexual well being, highlighted by looking at the team members roles are further reinforced by audit results to be presented in a separate paper where the patients treated by the participating study teams were asked to give their views on the information they had received during their care for cancer.

In terms of role clarity the surgeons, oncologists, radiologists and clinical nurse specialists information-giving roles appear to be well defined and understood by other team members. Importantly, there was good agreement within each of these specialities regarding the information regularly given to patients and colleagues' expectations also matched these views. However, agreement was less consistent when the roles of other specialities were reviewed. For example, in both the research and clinic nurse roles there was less than total agreement within each speciality as to the areas they regularly cover with patients and in conjunction with this other team members did not appear to have a consistent view of what these roles entail, similar role ambiguity has previously been described within breast care teams (Jenkins *et al*, 2001). Discrepancies between the information individuals' cover within a speciality or between that covered by a professional group and team perceptions could be due to a number of factors. For example, the healthcare professional may not feel confident that other team players cover the topic adequately, or it may reflect an attempt by an individual to compensate for uncertainty about their own role by trying to cover all the areas. Some discrepancies will reflect the team having insufficient understanding of a particular profession and this may be more likely with newer subspecialities or professions that are evolving with advances in treatments and technologies. Finally, patients regularly attempting to source information from a team member who would not usually cover a particular topic could force them in to incorporating it into their role while others in their speciality or colleagues in the team do not consider it part of the role. Unchecked these situations can lead to burnout in some individuals or feelings of being undervalued and can result in discontentment, ill will and poor staff morale and certainly there is evidence of this in the current study. There is also the potential for the provision of contradictory information being given to patients about their tests and treatments if team members have little idea about what their colleagues are covering.

The incidence of probable minor psychiatric morbidity overall (18%) is identical to the UK general population (Taylor *et al*, 1995) and marginally above that reported across 72 breast cancer teams (Haward *et al*, 2003) in which the mental well being of team members appears significantly better than in other studies of cancer clinicians (Ramirez *et al*, 1996; Taylor *et al*, 2005) and NHS staff in general (Wall *et al*, 1997). However, the current study only partially fits this pattern. Averaging across the teams gives a different picture to that represented by a team breakdown. Half the current sample has a case rate in a range 22–27% which is similar to previous data for other healthcare teams (Mullarky *et al*, 1999), NHS staff (Wall *et al*, 1997) and cancer clinicians (Ramirez *et al*, 1996) and one that is above the general population rate (18% – Taylor *et al*, 1995). The remaining teams have substantially lower case rates in a range 5–11%, which is even below the most recent rate reported for breast cancer teams (15.7% – Haward *et al*, 2003). The experience and effect of team working in these relatively new multidisciplinary configurations was the aim of the current study therefore it is important to appreciate that only some of the cancer teams have a majority of members enjoying good mental health and this is reflected in the MBI results too.

The overall prevalence of high levels of emotional exhaustion and depersonalisation from the subscales of the MBI (27 and 19%, respectively) in the current study are below all previously published rates; US medical staff – rates of 33% for each subscale (Maslach *et al*, 1996), less than rates of 32 and 24%, respectively, for UK consultants (Ramirez *et al*, 1996), and (for emotional exhaustion only) below the 40% rate most recently reported at follow-up in the same cohort of UK consultants (Taylor *et al*, 2005). The overall incidence of 31% for feelings of low personal accomplishment is just below the US norm but considerably lower than Ramirez *et al*'s (1996) UK data of 39%. However, breakdown by team clearly shows half of the teams have rates (range 35–50%) of high emotional exhaustion greater than the previous findings, while four out of the remaining five teams are markedly lower (range 7–13%). This pattern is repeated for feelings of low personal accomplishment with half of the teams showing rates (range 40–56%) greater than the previous findings while the remaining teams have rates (11–16%) considerably lower than the past data. The incidence of high depersonalisation shows only two teams have rates above the previous findings.

The current study revealed a fifth or less (range 13–22%) within each professional group scored above threshold on the GHQ-12, which is below previously published findings (Ramirez *et al*, 1996; Taylor *et al*, 2005) for some of these specialties. Previous samples (Ramirez *et al*, 1995, 1996; Taylor *et al*, 2005) have also demonstrated the stresses and strains of various professions in terms of burnout on the MBI subscales, notably high rates of emotional exhaustion in clinical and surgical oncologists. Our study found team leaders (who were invariably surgeons) and the nurses appeared prone to high levels of burnout on the emotional exhaustion subscale while it was the radiographers who had the greatest proportion (40%) reporting high levels of depersonalisation. Feelings of low levels of personal accomplishment were a pervasive problem for the histopathologists (69%) and a considerable proportion of the radiologists (43%).

It seems reasonable to assume that effective MDT working will provide benefits to patients and healthcare professionals working in cancer teams. Evidence has started to accrue showing that certain outcomes are improved (Landheer *et al*, 2001; Shankar *et al*, 2001; Van Laethem *et al*, 2001; Blumberg and Ramanathan, 2002, Soriano *et al*, 2002; Haward *et al*, 2003) but the current study suggests benefits are not always consistent, that advantages do not inevitably result from the adoption of a multidisciplinary approach and that there is room for improvement. We believe more research is needed to better understand how to gain the most from MDT working in cancer. Trusts may need to invest substantial resources in team training to ensure effective teamwork in order to reap the benefits for patients and healthcare professionals.

## Figures and Tables

**Table 1:** Team composition

**Teams**	**A**	**B**	**C**	**D**	**E**	**F**	**G**	**H**	**I**	**J**	**Total**
Surgeons	3	4	3	3	3	2	2	2	2	3	27
Gastroenterologists	2									1	3
Oncologists	1	1	3	a	4	2	2	2	1	1	17
Radiologists	2	5	3	3	1	1	2	1	2	3	23
Radiographers				1		4					5
Histopathologists	2	2	2	3	1	2	1	1	3	1	18
Breast physicians			1			2					3
Clinical nurse specialists	1	1	5	4	3	3	1	1	2	3	24
Clinic nurses	1					2					3
Chemotherapy nurse					1						1
Stoma care nurses	1	1								2	4
Palliative care physicians	1	3								1	5
Palliative care nurses	1	1						1			3
Research nurses			2	1		1					4
MDT coordinator			1					1		1	3
Clinical psychologist			1								1

Total	15	18	21	15	13	19	8	9	10	16	144

a Team D had one oncologist who declined to participate.

**Table 2a:** Information roles speciality *vs* colleagues view

	**Surgeon role**		**Oncologist role**		**Radiologist role**		**Clinical nurse specialist role**	
	**aSpeciality view**	**bColleagues view**	**Speciality view**	**Colleagues view**	**Speciality view**	**Colleagues view**	**Speciality view**	**Colleagues view**
Total *n*	27	110	17	121	23	117	23	113
Missing data		7		6		4	1	7
Explaining tests	27 (100%)	108 (98%)	11 (65%)	85 (70%)	21 (91%)	97 (83%)	19 (83%)	103 (91%)
Giving test results	27 (100%)	107 (97%)	13 (76%)	91 (74%)	14 (61%)	73 (62%)	18 (78%)	93 (82%)
Surgery	27 (100%)	108 (98%)	3 (18%)	33 (27%)	3 (13%)	12 (10%)	20 (87%)	102 (90%)
Radiotherapy	20 (74%)	80 (73%)	15 (88%)	117 (97%)	0	10 (9%)	21 (91%)	87 (77%)
Chemotherapy	21 (78%)	79 (72%)	17 (100%)	116 (96%)	0	6 (5%)	23 (100%)	91 (81%)
Hormone therapy (applies to breast teams only)	13/13 (100%)	55/62 (89%)	10/10 (100%)	61/66 (92%)	0/10	0/66	16/16 (100%)	51/59 (86%)
Prognosis	27 (100%)	106 (96%)	17 (100%)	115 (95%)	3 (13%)	10 (9%)	17 (74%)	75 (66%)
Clinical trials	17 (63%)	78 (71%)	15 (88%)	106 (88%)	3 (13%)	12 (10%)	13 (57%)	62 (55%)
Family history	25 (93%)	101 (92%)	14 (82%)	84 (69%)	1 (4%)	10 (9%)	15 (65%)	81 (72%)
Physical WB	24 (89%)	98 (89%)	16 (94%)	108 (89%)	9 (39%)	18 (15%)	23 (100%)	109 (96%)
Functional WB	16 (59%)	80 (73%)	14 (82%)	88 (73%)	3 (13%)	8 (7%)	23 (100%)	104 (92%)
Sexual WB	10 (37%)	48 (44%)	8 (47%)	49 (40%)	1 (4%)	0	19 (83%)	96 (85%)
Social WB	13 (48%)	49 (45%)	10 (59%)	61 (50%)	1 (4%)	2 (2%)	23 (100%)	103 (91%)
Emotional WB	14 (52%)	59 (54%)	10 (59%)	80 (66%)	2 (9%)	8 (7%)	23 (100%)	105 (93%)
Information leaflets (data for team C missing in colleagues' view)	9 (33%)	39/93 (42%)	15 (88%)	52/104 (50%)	5 (17%)	17/99 (17%)	23 (100%)	96/98 (98%)

a Speciality view=number of the speciality that reports giving information about the area.

b Colleagues view=number of colleagues that believe the speciality gives information about the area.

**Table 2b:** Information roles-speciality versus colleagues view

	**Research nurse role**		**Clinic nurse role**		**Stoma care nurse role**		**Palliative care nurse role**	
	**aSpeciality view**	**bColleagues view**	**Speciality view**	**Colleagues view**	**Speciality view**	**Colleagues view**	**Speciality view**	**Colleagues view**
Total *n*	4	46	3	29	4	41	3	33
Missing data		5		2		4		6
Explaining tests	2 (50%)	13 (28%)	2 (67%)	8 (28%)	2 (50%)	21 (51%)	1 (33%)	18 (55%)
Giving test results	2 (50%)	12 (26%)	1 (33%)	3 (10%)	2 (50%)	21 (51%)	1 (33%)	19 (58%)
Surgery	0	4 (9%)	2 (67%)	4 (14%)	4 (100%)	24 (59%)	1 (33%)	17 (52%)
Radiotherapy	3 (75%)	7 (15%)	1 (33%)	2 (7%)	3 (75%)	15 (37%)	2 (67%)	23 (70%)
Chemotherapy	3 (75%)	17 (37%)	1 (33%)	4 (14%)	3 (75%)	18 (44%)	2 (67%)	24 (73%)
Hormone therapy (applies to breast teams only)	3 (75%)	11/46 (24%)	2/2 (100%)	2/17 (12%)	N/A	N/A	N/A	N/A
Prognosis	1 (25%)	4 (9%)	1 (33%)	1 (3%)	2 (50%)	18 (44%)	3 (100%)	28 (85%)
Clinical trials	4 (100%)	39 (85%)	1 (33%)	1 (3%)	1 (25%)	13 (32%)	0	13 (39%)
Family history	0	5 (11%)	0	2 (7%)	3 (75%)	19 (46%)	2 (67%)	15 (45%)
Physical WB	3 (75%)	20 (43%)	3 (100%)	10 (34%)	4 (100%)	30 (73%)	3 (100%)	31 (94%)
Functional WB	2 (50%)	16 (35%)	3 (100%)	8 (28%)	4 (100%)	29 (71%)	3 (100%)	31 (94%)
Sexual WB	1 (25%)	10 (22%)	1 (33%)	4 (14%)	4 (100%)	31 (76%)	2 (67%)	29 (88%)
Social WB	1 (25%)	12 (26%)	3 (100%)	5 (17%)	4 (100%)	30 (73%)	3 (100%)	31 (94%)
Emotional WB	3 (75%)	13 (28%)	3 (100%)	7 (24%)	4 (100%)	29 (71%)	3 (100%)	31 (94%)
Information leaflets (data for team C missing in colleagues' view)	3 (75%)	14/29 (48%)	3 (100%)	9/29 (31%)	4 (100%)	32/41 (78%)	3 (100%)	26/33 (79%)

N/A=not applicable.

a Speciality view=number of the speciality that reports giving information about the area.

b Colleagues view=number of colleagues that believe the speciality gives information about the area.

**Table 3:** Number of professions out of the 14 specialties represented across the 10 teams regularly^a^ covering each information area

**Information area**	**Professional groups regularly providing information**	***n***
Explaining tests	Surgeon, radiologist, CNS,^b^ radiographer, palliative care physician, gastroenterologist, breast physician	7
Giving test results	Surgeon, oncologist, CNS, palliative care physician, gastroenterologist, breast physician	6
Surgery	Surgeon, CNS, stoma care nurse	3
Radiotherapy	Oncologist, CNS, research nurse, stoma care nurse, chemo nurse	5
Chemotherapy	Surgeon, oncologist, CNS, research nurse, stoma care nurse, chemo nurse	6
Hormone therapy (breast teams only)	Surgeon, oncologist, CNS, research nurse, clinic nurse, breast physician, chemo nurse	7
Prognosis	Surgeon, oncologist, palliative care physician, palliative care nurse, gastroenterologist, chemo nurse	6
Clinical trials	Oncologist, research nurse, chemo nurse	3
Family history	Surgeon, oncologist, palliative care physician, stoma care nurse	4
Physical well being	Surgeon, oncologist, CNS, clinic nurse, research nurse, palliative care physician, stoma care nurse, palliative care nurse, gastroenterologist, breast physician, chemo nurse, clinical psychologist	12
Functional well being	Oncologist, CNS, clinic nurse, palliative care physician, stoma care nurse, palliative care nurse, gastroenterologist, chemo nurse, clinical psychologist	9
Sexual well being	CNS, palliative care physician, stoma care nurse, chemo nurse, clinical psychologist	5
Social well being	CNS, clinic nurse, palliative care physician, stoma care nurse, palliative care nurse, gastroenterologist, chemo nurse, clinical psychologist	8
Emotional well being	CNS, research nurse, clinic nurse, palliative care physician, stoma care nurse, palliative care nurse, gastroenterologist, chemo nurse, clinical psychologist	9
Information leaflets	Oncologist, CNS, research nurse, clinic nurse, stoma care nurse, palliative care nurse, chemo nurse	7

a Regularly is defined as ⩾75% of members of the professional group agree they provide information on the area.

b CNS denotes the clinical nurse specialist.

**Table 4:** Distribution of GHQ-12 caseness and high ‘burnout’ levels on the MBI subscales by team and by speciality^a^

			**MBI subscales**		
		**GHQ-12 ⩾4**	**EE ⩾27**	**DP ⩾10**	**PA ⩽33**
	**Total *n***	***n* (%)**	***n* (%)**	***n* (%)**	***n* (%)**
Total sample	144	25 (18^c^)	39 (27^c^)	27 (19^b^)	44 (31^d^)
*By team*					
Team A	15	1 (7^b^)	1 (7)	0	2 (13)
Team B	18	4 (22)	5 (28)	3 (17)	10 (56)
Team C	21	1 (5)	7 (35^b^)	5 (25^b^)	9 (45^b^)
Team D	15	4 (27)	7 (47)	8 (53)	6 (43^b^)
Team E	13	1 (8)	1 (8^b^)	1 (8)	6 (50^b^)
Team F	19	5 (26)	7 (37)	3 (16)	3 (16)
Team G	8	2 (25)	4 (50)	3 (38)	1 (13)
Team H	9	2 (22)	4 (44)	2 (22)	1 (11)
Team I	10	1 (11^b^)	1 (10)	2 (20)	4 (40)
Team J	16	4 (25)	2 (13)	0	2 (13)
					
*By speciaility*					
Team leaders	10	2 (20)	4 (40)	3 (30)	3 (30)
Surgeons	27	4 (15)	6 (22)	8 (30)	8 (30)
Oncologists	17	2 (13^b^)	5 (29)	5 (29)	5 (29)
Radiologists	23	4 (17)	5 (22)	3 (13)	10 (43)
Histopathologists	18	4 (22)	5 (29^b^)	5 (28)	11 (69^c^)
Palliative care physicians	5	0	1 (20)	0	1 (17)
Nurses	39	8 (21)	13 (33)	4 (10)	7 (18)
Radiographers	5	1 (20)	1 (20)	2 (40)	0

EE=emotional exhaustion, DP=depersonalisation, PA=personal accomplishment.

a To maintain anonymity data are not presented for specialties with ⩽3 members.

b one missing data.

c Two missing data.

d Three missing data.
